# Cancer Stem Cells: Cellular Plasticity, Niche, and its Clinical Relevance

**DOI:** 10.4172/2157-7633.1000363

**Published:** 2016-10-26

**Authors:** Gina Lee, Robert R Hall, Atique U Ahmed

**Affiliations:** Department of Neurological Surgery, Northwestern University, Chicago, Illinois, USA

**Keywords:** Cancer stem cells, Tumor cells, Niche, Cellular plasticity

## Abstract

Cancer handles an estimated 7.6 million deaths worldwide per annum. A recent theory focuses on the role Cancer Stem Cells (CSCs) in driving tumorigenesis and disease progression. This theory hypothesizes that a population of the tumor cell with similar functional and phenotypic characteristics as normal tissue stem cells are responsible for formation and advancement of many human cancers. The CSCs subpopulation can differentiate into non-CSC tumor cells and promote phenotypic and functional heterogeneity within the tumor. The presence of CSCs has been reported in a number of human cancers including blood, breast, brain, colon, lung, pancreas prostate and liver. Although the origin of CSCs remains a mystery, recent reports suggest that the phenotypic characteristics of CSCs may be plastic and are influenced by the microenvironment specific for the individual tumor. Such factors unique to each tumor preserve the dynamic balance between CSCs to non-CSCs cell fate, as well as maintain the proper equilibrium. Alternating such equilibrium via dedifferentiation can result in aggressiveness, as CSCs are considered to be more resistant to the conventional cancer treatments of chemotherapy and radiation. Understanding how the tumoral microenvironment affects the plasticity driven CSC niche will be critical for developing a more effective treatment for cancer by eliminating its aggressive and recurring nature that now is believed to be perpetuated by CSCs.

## Introduction

Multicellular organisms require a poised homeostatic equilibrium between cellular proliferation and differentiation for development and growth of the individual. Disruption of this equilibrium causes the devastating consequences of malignancy. A recently proposed hierarchical model of tumorigenesis postulates that the uncontrolled expansion of many cancers is predominantly driven by a rare subset of cells within the tumor population known as Cancer Stem Cells (CSCs) [1,2]. Although similar to somatic stem cells, CSCs have an enhanced potential to self-renew, differentiate into non-stem cancer cells and promote intratumoral heterogeneity in order to sustain uncontrolled tumor growth. It has also been reported that CSCs have an innate ability to resist conventional multi-modality therapy and considered to be partly responsible for the high rate of disease recurrence and clinical relapse observed in many cancers [3,4]. Recent evidence suggests that therapeutic stress may also promote cellular plasticity, which mediates the conversion of normal cancer cells to a CSC-like state [5,6]. These newly converted stem-like cells possess enhanced tumor formation abilities and are more infiltrative than non-stem cancer cells in the animal model, adding to the attenuated therapeutic efficacy seen in clinical settings. The data argues against the unidirectional flow of cellular hierarchy, rather suggesting a bidirectional flow whose activation may be influenced by various factors within the tumor-specific microenvironment or "niche" [7,8]. The dynamic equilibrium between CSCs and their lineage-committed non-stem counterparts is partly regulated by the rate of differentiation and the balance between asymmetric and symmetric cell division in the CSC compartment. Because the heterogeneous tumor population contains a small number of CSCs amid the larger number of non-stem differentiated tumor cells, it is essential to understand the regulation of such equilibrium. Any shift in the equilibrium state will critically influence the clinical outcomes and lead to a more CSC-rich tumor, which will be more aggressive and produce poorer prognoses in patients [9,10]. By elucidating the various mechanisms for the maintenance of this equilibrium state and the relationship between Cancer Stem Cells and their niche, one can improve the current standard of care as well as develop targeted strategies that will enhance the therapeutic efficacies of anti-cancer therapies. In this recent review, we will summarize the recent finding of the mechanisms of the intratumoral cell fate equilibrium and the consequence of its regulation in disease progression, as well as discussing the potential development of therapeutic modalities that target CSCs.

## The CSC Hypothesis and its Role in Disease Progression and Tumorigenesis

Whereas normal tissues display an ordered developmental structure underlying cellular heterogeneity, allowing various cell types to maintain the generation of stable differentiated progeny cells through epigenetic regulation, malignant tissues possess disorganized cellular programming that gives rise to heterogeneous cancer cell populations [11]. In attempt to explain the development of heterogeneous tumors, two competing theories have been proposed: the clonal evolution theory and the cancer stem cell theory. In 1976, Peter Nowell first proposed the clonal evolution model and introduced the idea that cancer was driven by the accumulation of somatic cell mutations [12,13]. This theory postulates that a single clone survives an oncogenic mutation that leads to a more aggressive phenotype, and upon proliferation, its daughter cells acquire additional somatic mutations that further promote survival fitness and aggressive behavior. The aggressive daughter cells continue to divide and eventually outnumber the non-aggressive populations because of their high fitness. In time, the clones acquire additional mutations, creating genetically diverse clonal populations. These clones evolve through what resembles Darwinian selection of survival of the fittest, in which only those clonal populations that can survive the accumulation of many mutations will comprise the final heterogeneous tumor [14]. According to the clonal evolution model, each cell within the tumor is considered to possess an equal potential to promote tumorigenesis, leading to the devastating outcomes of human malignancy. This concept is the foundation of a majority of the currently available cancer therapies, which are designed to target and eliminate all the cancer cells within a tumor with higher proliferative capacity to achieve cures. However, in many human malignancies, especially solid cancers, ‘cures' remain relatively rare commodities and cancer-related death rates remain very high [15].

The more recently proposed hierarchical model of tumorigenesis claims that only a rare subset of cells, known as Cancer Stem Cells (CSCs), is responsible for the uninhibited tumorigenic capacity of malignant cancers. Although similar to normal stem cells, CSCs have an enhanced potential to self-renew, differentiate into non-stem cancer cells, and initiate new tumor formation by giving rise to heterogeneous cell populations [4,16,17]. In contrast to the clonal evolution model, multipotent characteristics of CSCs are considered to be responsible for intratumoral heterogeneity through their aberrant differentiation capacity [18]. The cells with the most tumorigenic potential exist at the top of a hierarchical organization that once closely resembled the ordered of developmental structure for normal tissues but has since become disordered [19]. These cells have also been shown to resist primary multi-modality therapies in some cancers, which contribute to the dismal prognoses in patients, with inevitable disease relapses [20,21]. According to the CSC model, the only way to prevent disease relapse and achieve durable therapeutic response is to eliminate the CSC population. While differentiated cancer cells do not have the ability to self-renew indefinitely and cannot produce cells of different origins, the enriched properties of CSCs allow the formation of diverse, heterogeneous tumor populations [22,23].

Melanoma is widely considered to strongly support the CSC model [24]. Initially, melanomas form flat lesions that can be removed by gross resection [25]. However, melanomas quickly progress to contain heterogeneous subpopulations that express different genes [26]. This property makes metastatic melanomas extremely difficult to treat because of their ability to mimic vasculature, a property that suggests the presence of non-differentiated cells in the tumor population [27]. Furthermore, malignant melanomas that exhibit higher expression of stem cell markers correlate with poor prognoses [28] and resistance to the cytotoxic agent doxorubicin [29]. These therapies eradicate most of the tumor growth, but populations of resistant cells remains, which give rise to novel tumors that are chemoresistant and immunoevasive [30]. Because melanomas lack homogeneous clones with similar genomic profiles and instead exhibit a hierarchical structure of mature and progenitor cells, melanoma supports the CSC model. On the other hand, some malignant tumors in other tissues seemingly contradict the CSC theory. Retinoblastoma can occur after only two mutations [31], a property that supports the clonal evolution theory. Examination of the karyotypes of retinoblastoma cells showed that non-disjunction in chromosome 13 was to blame for this malignancy, further supporting the idea that sequential mutations in identical cells drive tumor development and progression [32]. As these cases exemplify, it is highly unlikely that one model of tumorigenesis is completely correct, but rather it is more likely that tumor development exhibits characteristics from both [33,34]. The two models can be used in conjunction to explain more effectively the basis of tumor heterogeneity, disease progression and recurrence.

**Figure 1:**
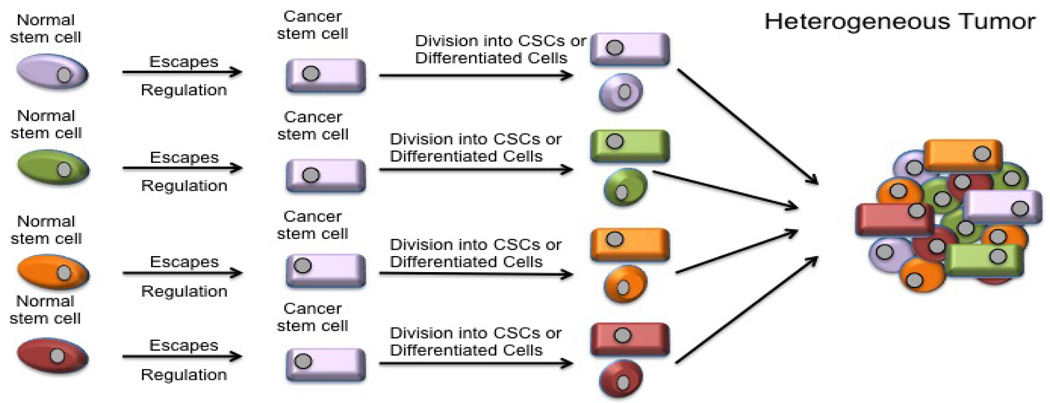
Hierarchy leading to heterogeneity. The CSC theory claims that CSCs arise from normal stem cells within the population and that only these CSCs drive tumorgenesis. These CSCS can either self renew or divide into differentiated progeny. Since the cells in a tumor come from different CSCs, the tumor is heterogenic.

Human cancers frequently display substantial heterogeneity with many phenotypic features such as cellular morphology, gene expression (including cell surface markers, growth factors, and hormonal receptors), metabolism, and angiogenic, immunogenic, and metastatic potentials [35]. The CSC model proposes that while every cell in the cancer population is genetically equal, individual subsets within the tumor possess internal clonal heterogeneity [34]. This model puts the most tumorigenic cells at the top of its proposed hierarchy. These cells can go through the asymmetric cellular division, which results in one differentiated less-tumorigenic or non-tumorigenic cancer cells, as well as inducing self-renewal of CSC daughter cells. It is these small CSC subpopulations that are believed to drive the initiation and expansion of the entire tumor, while non-CSCs, which constitute the majority of the tumor, contribute much less to tumorigenesis and growth, instead influencing the overall traits of established tumors (Figure 1) [34].

One key factor that drives the regulation of tumor growth and heterogeneity is genetic instability, which results from an increased rate of cell proliferation in addition to mutations and epigenetic alterations [11]. If the cell cycle checkpoint fails to repair an error (i.e. additional or insufficient number of chromosomes or mutations) during replication, the cell will progress to the next step in the cycle, wreaking further havoc on genomic stability [36]. Both normal cells and cancer cells show spontaneous mutations and are usually targeted by DNA checkpoint and repair mechanisms to prevent the accumulation of aberrant cells. However, some mutations alter gene function in the DNA repair pathway and push cells to respond to the internal and external pressures from Darwinian selection [11]. The cells that thrive despite these genetic changes can evade DNA checkpoints and immunosurveillance to survive longer and eventually give rise to descendant cells hosting the same or added genomic abnormalities.

## Cellular Plasticity

**Figure 2:**
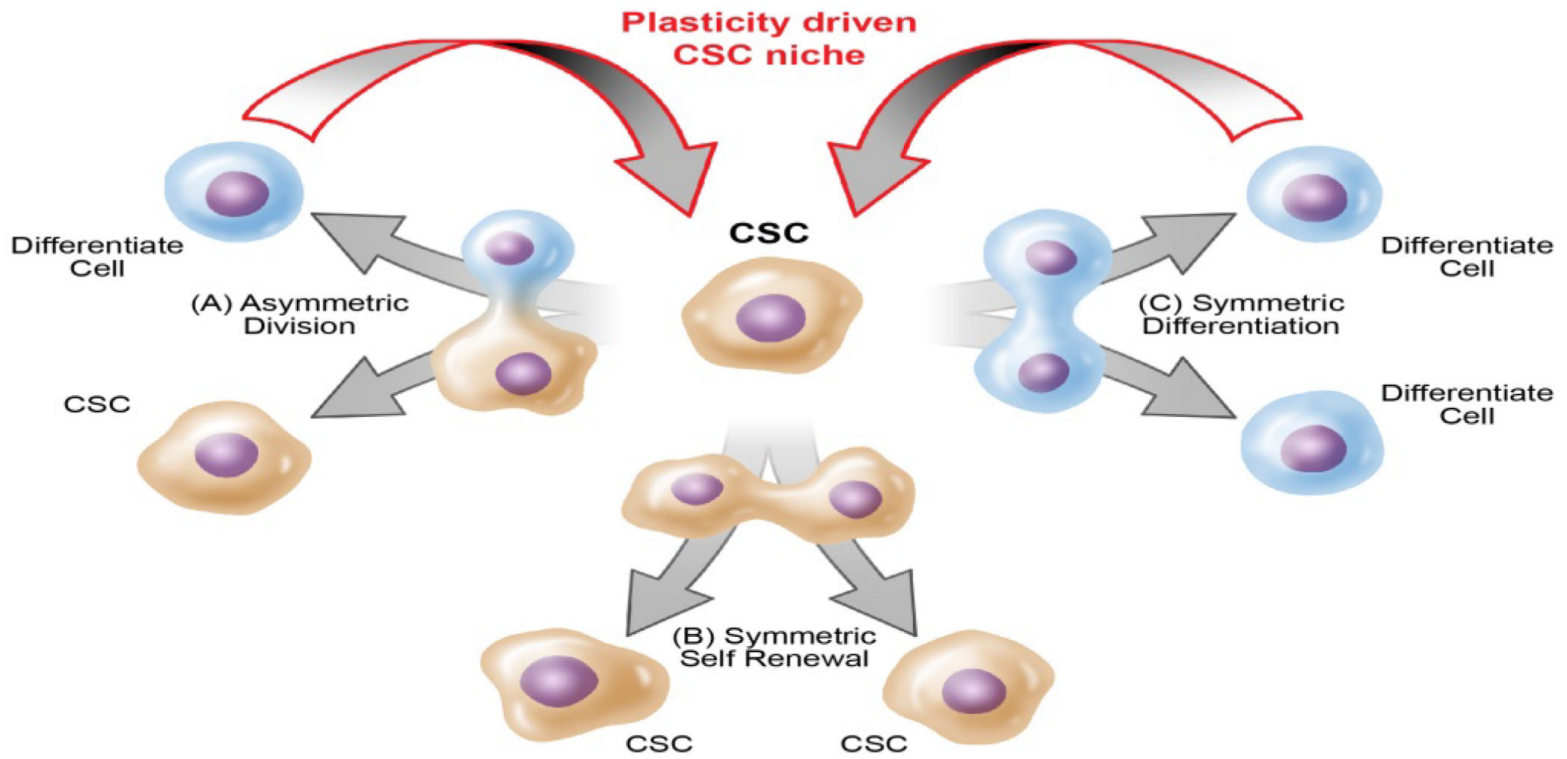
Asymmetric division of CSCs. (A) Asymmetric cellular division yields one undifferentiated CSC and one differentiated cell. Under normal conditions, this is the second most common division, but during TMZ treatment this type of division occurs least. (B) Symmetric cell renewal produces two daughter CSCs that are identical to the mother CSC. Under normal conditions, this is the least observed division, but becomes the most prevalent when the population is treated with TMZ. This largely contributes to the resistance and reoccurrence of tumors. (C) Symmetric differentiation yields two differentiated progeny. Under normal conditions, this division occurs most frequently. However, during TMZ treatment, this division is seen second most often.

The proper development of a multicellular organism depends on the balanced equilibrium between differentiated cells committed to tissue lineages and cells with stem-like characteristics. The concept that CSCs and their more differentiated progeny exist in a dynamic equilibrium state has been recently proposed by many labs, including our own. These reports not only showed that CSCs can differentiate to possess a committed fate, but that non-stem-like cells can also acquire CSC-like state [6,9,19,37,38], which results in a dynamic relationship between the two populations. To maintain this intrinsic homeostatic state, a stable balance between the rates of CSC self-renewal, differentiation, and asymmetric division and the rates of interconversion between non-CSCs to CSCs must be maintained within the individual tumor [39]. One critical aspect of normal stem cell function and identity is asymmetric division, in which the cell creates descendants that retain the parental stemness characteristics as well as give rise to progeny cells that are committed to differentiation into multiple lineages [40]. The symmetrical division is also a fundamental feature of the tissue generation of normal adult stem cells in which two daughter cells preserve their stemness. These tightly controlled processes of cellular division are required to maintain dynamic balance and diversity among cell types and contribute to the global size of the tissue or organism (Figure 2). Any disruption of this process throws off the equilibrium ratio of number of stem cells to non-stem cells. The dedifferentiation of non-CSCs into CSCs has been attributed to cellular reprogramming during oncogenic transformations and initiates subsequent development of aggressive cancers [3,6,9,40,41]. These unprecedented rates of plasticity allow normal cancer cells to acquire stem-like states and in this way increase the intratumoral CSC frequency.

Normal developmental processes allow for the differentiation of progenitor cells, and because of cellular plasticity, these cells can adopt new and differentiated fates. Cellular plasticity is defined as the ability of adult tissue cells to undergo a dedifferentiation or differentiation process to adopt new phenotypic and functional identities [34,42,43]. During normal development, the change from stem cell to lineage-committed cell is a gradual process with phases that cause the cell to lose developmental potential until it reaches its final committed, differentiated state. At this point, we can see that stem cells exist in a dynamic equilibrium with their differentiated counterparts in a stable balance that is tightly regulated by various signaling pathways associated with microenvironments and external stimuli. When this balance is disturbed by transcriptional, epigenetic, or environmental changes, non-CSCs can undergo a dedifferentiation process to acquire stem-like characteristics and be reprogrammed towards a more aggressive tumorigenic fate [43]. Given the possible significance of this equilibrium process, next we will discuss various factors that can influence the intratumoral cell fate state.

## The CSC Niche and Microenvironment in Disease Progression

Niches are pockets of distinct microenvironment with specific functional characteristics that form the habitat of certain cells with specific fates. Such microenvironmental pockets are regulated by a variety of factors and cell types, including immune cells, cancer-associated fibroblasts, extracellular matrix components, hypoxia, and pH [8,43–45]. Cells found in the CSC niche are capable of maintaining or even acquiring stem-like characteristics to promote the fitness of each tumor [18,46,47]. A critical unanswered question is whether any cell that encounters this niche is capable of initiating the cellular reprogramming process to acquire a stem-like state or if only the cells with stem or progenitor cell state/lineage are capable of such reprogramming. In light of recent discoveries about cellular reprogramming both in the developmental as well as pathological context, it is conceivable that any cell in the presence of appropriate signals is capable of initiating the cellular reprogramming process to acquire the stem-like state. The example of fate reprogramming is also reported in the normal developmental setting, as certain progenitor populations demonstrate the ability to dedifferentiate to acquire stem-like characteristics. In Drosophila testes, a cluster of stromal cells known as spematogonial can dedifferentiate into germline stem cells to replace stem cell populations in the aging tissue [48]. This dedifferentiation process is also observed in the mouse testes [49]. In the hematopoietic system, PAX5 has been shown to be crucial in the differentiation process of lymphoid progenitor cells to mature B-cells [50]. A report showed that the deletion of PAX5 led to the dedifferentiation of a mature B-cell into a T-cell [51]. Thus, the elimination of cellular identity through a strong master regulator such as PAX5 can achieve reprogramming. The process of fate reprograming is also supported by landmark study published by the Yamanaka's lab in 2006, where they showed that reprogramming committed cells back to an induced pluripotent state required the stimulation of four transcription factors: Sox2, Oct4, Klf4, and c-Myc [15]. Authors showed that cells resting in a quiescent developmental state could be reprogrammed to become pluripotent progenitor cells by introduction of these factors. These four factors are involved in a poorly understood network comprised of other transcription factors, histone modification enzymes, and polycomb group complexes that collectively capable of reprograming of differentiated cells [52].

**Figure 3:**
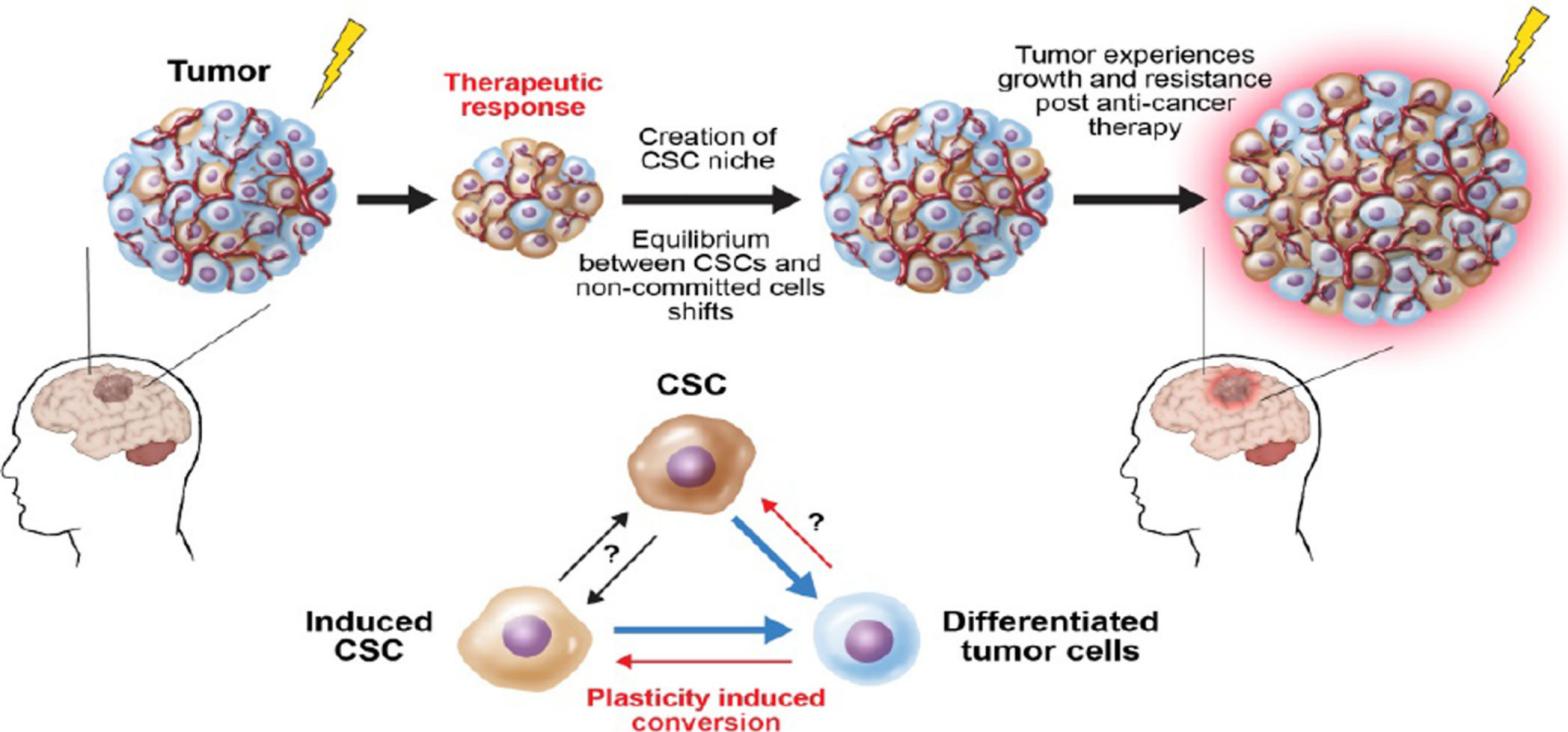
CSC, microenvironment and intratumoral equilibrium. When a tumor is exposed to hypoxia, low pH, chemotherapy or radiation, a microenvironment that favors CSCs is created. Because of this, some mature cells in the tumor dedifferentiate and stemness in the present CSCs is maintained. This plasticity of the tumor leads to drug resistance and disease reoccurrence.

In the pathological condition, recent evidence widely supports the theory describing spontaneous and therapy-induced reprogramming of differentiated cancer cells to cancer stem-like cells. Iliopoulos et al. showed that IL6 was one of the key factors that mediated the conversion of non-CSCs to CSCs in breast cancer and prostate cancer [9]. They used a chemotherapy-induced model of oncogenesis to indicate that both non-CSCs and CSCs exist in a dynamic equilibrium in which, over many generations, the proportion of these two cell subpopulations remain continuous. A similar shift in the cell fate equilibrium is also present in Glioblastoma Multiforme (GBM), as differentiated glioma cells converted to glioma stem-like cells after exposure to clinically relevant doses of primary chemotherapy [6]. It is shown that this chemotherapy-induced cellular plasticity can enhance the Glioma Stem Cells (GSC) subpopulation in recurrent tumors and may be responsible for a more aggressive tumor phenotype [3,21]. During disease progression, the tumor-specific niche creates a dynamic equilibrium between the CSCs and their fate committed counterparts [2,9,53]. Any shift in this equilibrium state is regulated by the intratumoral microenvironment and can potentially influence the clinical outcomes of tumors since CSCs possess the intrinsic ability to resist conventional therapies (Figure 3). For the majority of solid cancers, the molecular mechanism of how this equilibrium is maintained with respect to a tumor-specific CSC niche continues to be poorly understood.

One proposed explanation for the maintenance of the CSC population is the Epithelial-to-Mesenchymal Transition (EMT) [54]. While this process is a key step in metastatic progression, it is also essential in development [55]. Recently, embryonic transcription factors have been shown to drive classic cancer traits, including apoptosis resistance [56] invasiveness [57] and motility [58]. The basal-like mammary cells in breast cancer have the ability to dedifferentiate spontaneously into a stem-like state post oncogenic transformation via EMT [10]. In GBM, CD95 has been shown to assist in the maintenance of EMT programming and to provide stem cell properties to glioma cells [59,60]. Overexpression of the transcription factor Twist, which is known to promote EMT, resulted in increased invasive potential and therapeutic resistance in two PDX glioma lines [61]. Notably, in GBM Twist did not induce the E-cadherin to N-cadherin switch that is observed in EMT in other tissues [61], suggesting that this factor promotes maintenance of stem cells in the brain rather than driving classical EMT.

In addition to tumor population hijacking properties that induce EMT in development to maintain CSC populations, the tumor microenvironment also promote the maintenance of CSCs. Because of their inability to access oxygen-containing vasculatures, tumors are more hypoxic than the surrounding healthy tissues [18]. It has been reported that hypoxia and intratumoral pH can promote a stemness niche by enriching an environment that supports the self-renewal capacity of stem cells through activation of various stemness associated genes and initiates dedifferentiation of non-CSCs [43,44,62,63]. Cellular response to hypoxia is commonly regulated by hypoxia inducible factors (HIFs), which are key transcriptional factors that are upregulated upon exposure to low-oxygenated conditions [64]. It has long been known that anti-cancer therapy can change the tumor microenvironment and induce therapeutic resistance in cells residing within this region [7,8]. We reported that anti-cancer therapy can induce these hypoxia-like responses to dedifferentiate non-CSCs to their stem-like state, and newly converted CSCs overexpressed both HIF1α and HIF2α [6]. Anti-glioma chemotherapy significantly enhances the number of intratumoral hypoxic foci in an orthotopic xenograft model and shifts the stemness equilibrium towards a more stem-like state. Thus, it is conceivable that hypoxia induced CSC niche may shift the intratumoral balance between CSCs and non-CSCs towards a more stem-like state, which in turn contributes to therapeutic resistance. The precise mechanism of hypoxia-induced CSC niche and its contribution to promoting therapeutic resistance still requires further investigation.

Hypoxic conditions also stimulate angiogenesis by inducing the release of angiogenic factors, including vascular endothelial growth factor (VEGF). Although HIF1α is often considered the master regulator of angiogenesis in hypoxia, HIF2α drives angiogenesis by regulating VEGF. VEGF has become one of the most well-known endothelial cytokines modulated by hypoxia, and its contribution to angiogenesis and tumor malignancy has been studied in various tumor models [65–67]. Angiogenesis and the production of a vascular network are essential for tumor and disease progression, and Stockmann et al. showed that VEGF was crucial for the formation of this vasculature. The perivascular niche that promotes maintenance of CSCs has been well documented. In the PDGFR-induced glioma model, nitric oxide-mediated expression of Nestin, Notch, and NO leads to stem-like characteristics in glioma cells, which enhance tumorigenic capacities *in vivo* [68]. The perivascular niche also induced VEGF expression, and a recent study has demonstrated that autocrine signaling of VEGF-VEGFR2 can promote GSC viability and tumor growth [69]. In a mouse model of skin cancer, efficient blocking of VEGFR2-neuropilin signaling can effectively deplete the CSC population [70]. Collectively, these studies point towards a notion that targeting the perivascular niche of CSCs by blocking VEGF-VEGFR2 signaling might be an effective CSC targeting strategy. Inhibition of VEGF function by using Bevacizumab, a humanized monoclonal antibody against VEGFA, has received accelerated approval to target tumor angiogenesis in glioma patients [71]. However, despite improving the quality of the patients' lives, nearly all patients with GBM progress, and Bevacizumab has been shown to enhance the dissemination characteristic in GBM [72]. Schnegg et al. recently demonstrated that VEGF-A inhibitors promote HIF1α-mediated expansion of the CSC population in melanoma, elegantly highlighting the role of therapy adaptive resistance mechanisms driven by the therapeutic stress induced selection pressure [73].

Angiogenesis can also be a result of a microenvironment with low pH, which has also been reported to contribute to the maintenance of the CSC niche [44]. For many cancers, extracellular pH levels are significantly more acidic than in normal tissues and are indirectly correlated to tumor size [44,74,75]. A shift to an acidic pH within the intratumoral microenvironment increases the expression of cancer stem cell markers and promotes the equilibrium to move towards stemness. CSCs exposed to an enriched therapy-induced stem cell niche will further increase cell proliferation, angiogenesis, immunosuppression, and chemoresistance [44,74–77], which contribute to the poor prognoses of many cancers. Such plasticity-mediated adaptability may be critical for cancer cells to overcome targeted anti-cancer therapies and promote therapeutic resistance. Elucidating the molecular mechanisms that govern cellular plasticity will allow the development of effective targeting strategies to eliminate newly developed CSCs.

## Clinical Implications of Cancer Stem Cells

One of the most difficult endeavors in the cancer stem cell field is to understand their contribution in the clinical setting. It is known that CSCs appear to be more resistant to conventional therapies such as radiation and chemotherapy than normal cancer cells because of their quiescence, or dormancy [78,79]. Tumor quiescence is a state of remission in which cancer cells are resting and undetectable for a period. This resting phase is commonly seen in patients who have endured constant multi-modal therapies such as radiation and chemotherapy [48,80,81], which contributes to the poor outcomes in the clinical setting (Figure 3). Here, we summarize the published results in attempt to understand the contribution of CSCs in the clinical setting and postulate how to exploit some unique CSC characteristics to develop novel anti-cancer therapies.

## Therapy-resistant CSCs in Disease Relapse

Disease relapse and tumor metastases are some of the major causes behind the unfortunate survival rates in cancer patients who, after a certain point during their course of treatment, fail to respond to conventional therapy. Enhanced therapeutic resistance has been attributed to CSCs [80,82–87], which subsequently leads to increased tumor growth, invasion, and relapse. A common contributor to therapeutic resistance is enhanced DNA damage response. Radiation, in addition to many current chemotherapy drugs (such as Cisplatin, Temozolomide, Methotrexate, and Doxorubicin), induces cell death by disrupting and damaging DNA. Because DNA is the genetic makeup of every cell in the human body, the inability to repair this damage is fatal to the cells [88,89]. The most lethal effect that these previously mentioned forms of therapy have on DNA is produced by double strand breaks. Double strand breaks are typically repaired through either homologous recombination or non-homologous end joining [85,90,91]. For a more detailed summary on homologous and nonhomologous DNA double strand break repair, refer to Cojoc et al. [85].

One particularly interesting DNA repair gene that has been associated with cancer in the context of therapeutic resistance is Rad51 [49,92,93]. During double strand break repair, a template strand will replace the missing base pairs by invading the paired strands of homologous DNA. Rad51 catalyzes the search for and the invasion of the homologous DNA strand as well as the repair initiation and the annealing of the double strand break [94]. Recent data has shown that Rad51 is overexpressed in CSCs and that this increase in expression may be responsible for the therapeutic resistance observed post-radiation and after primary chemotherapy [49,95–98]. Furthermore, the inhibition of Rad51 promoted the resensitization of these previously resistant cells to anti-cancer therapy [99–101], which suggests that enhanced DNA repair activity in CSCs promotes their ability to overcome any double strand breaks during therapy.

## Targeting the CSC Niche for Anti-cancer Therapy

Conventional chemotherapy, which includes any combination consisting of surgery, radiation, and chemotherapy, is currently the main form of treatment for cancer patients. However, there are a number of cancers that quickly become drug resistant and cause disease relapse or tumor metastases. Because CSCs have been shown to have a higher potential to resist conventional therapy and also possesses inherent self-renewal properties, it is crucial to develop strategies that will target these aggressive populations. The intratumoral microenvironment retains the ability to enrich and initiate stemness in cancer cells, so by preventing cellular plasticity through stem niche factors, the CSC phenotype can also be diminished. As previously mentioned, one tumor microenvironmental factor that has been intensively studied is hypoxia. Targeting hypoxia can manipulate the CSC niche, and both HIF1α and HIF2α have been shown to be promising therapeutic targets in glioma [18,102,103]. Anti-angiogenic therapy can also diminish the tumor vasculature as well as inhibit the self-renewing capacities of CSCs [46,104]. Inhibiting angiogenic factors will lead to the starvation of tumors, further inhibiting tumor growth and proliferation of CSCs. However, a pre-clinical study by Schnegg et al. indicated a possible cellular plasticity mediated enrichment of CSCs post anti-VEGF therapy and thus may require further optimization of such therapeutic approaches [73].

Also, to targeting the stem cell niche, increased expression of Rad51 in cells with CSC-like phenotypes has been associated with chemoresistance [49,95]. The enhanced ability of CSCs to repair double-strand breaks in their DNA allows them to overcome damage made post-radiation and/or chemotherapy. Recent publications show that inhibiting Rad51 expression, specifically in the CSC subpopulation, causes once-resistant tumors to become re-sensitized to therapy [99–101]. There has also been an increase in the search for new drugs that can directly target and kill CSCs by inducing toxicity, inhibiting self-renewal, or sensitizing them to future therapy [64,105–108]. However, being able to target CSCs has been an incredibly difficult hurdle to overcome due to the inherent plastic nature of tumor cells to become CSC-like. A promising strategy includes the differentiation of these CSCs to adopt a more benign, lineage-committed fate so they will have a more therapy-sensitive, less aggressive and invasive phenotype. By constantly inducing the differentiation of CSCs, they can be targeted without the challenge of cells reverting to their stem-like state. It is known that bone morphogenetic proteins (BMPs) initiate differentiation of stem cells and can result in decreases in proliferation, tumor growth and initiation [109–115]. BMP administration has been combined with currently existing chemotherapy [116–121], and by doing so, the dynamic conversion of non-CSCs to cells possessing a therapy resistance CSC-like phenotype can be targeted to enhance therapeutic sensitivity as well as efficacy (Table 1).

## Conclusion

A recent model of tumorigenesis states that a small subset of cells within the cancer population, known as Cancer Stem Cells, is responsible for the initiation and expansion of tumors. Cells with this stem-like phenotype possess a higher potential for self-renewal and tumor formation and have also been shown to be more resistant to conventional therapies. CSCs exist in a dynamic equilibrium with non-CSCs, and any shift in this balance will potentiate the negative effects of cancer. Although it is known that CSCs can differentiate to have lineage-committed fates, it has now been postulated that these differentiated cells can dedifferentiated and adopt CSC-like phenotypes. Conventional therapies can further enhance the frequency of plasticity as the tumor microenvironment continues to change and pressure the equilibrium to shift, favoring the survival of CSCs. To prevent the aggressive, invasive, and resistant nature of the CSCs, it is crucial to better understand both CSC biology and their niche within tumors. It is not clear if the current stem cell model can account for such reversible intraconvertibility. If the frequency of such conversion is low, it may possible to distinguish the newly converted CSCs and incorporate their biological effects in the current model. However, in light of recent reports that demonstrate that such conversion is significantly accelerated post oncogenic transformation and during anti-cancer therapies, the current CSC model may be unfit for this condition. New models may be required to describe the plastic behavior of human cancer [5,6,10]. Elucidating the mechanisms that drive cellular plasticity and exploring how intratumoral microenvironmental changes affect plastic behavior of cancer cells can further enhance clinical efficacy of current therapies to benefit the many patients and families affected by human malignancies.

## Figures and Tables

**Table 1:** Recent findings depict the clinical relevance of CSCs and suggest novel treatment modalities to shrink the CSC population and reduce the inavsive and therapeutic resistant properties of tumors.

Finding	Significance	Reference
CSCs are senescent	Dormancy allows CSCs to resist conventional cancer treatments.	[78,79]
CSCs overexpress Rad51	Rad51 assists in DNA repair following double strand breaks. Overexpressing Rad51 allows CSCs to repair damage caused by chemotherapy and radiation.	[49,95–98]
BMPs induce differentiation in stem cells	Treating CSCs with BMPs may initiate differentiation and thus decrease proliferation and tumor growth.	[116–121]
Self-renewal of CSCs begets chemoresistance	Therapies that prompt CSCs to adopt committed states may reduce aggression, invasive capabilities and therapeutic resistance.	[109–115]
Hypoxia induces expansion of the CSC population	Targeting HIF1α and HIF2α with inhibitors may halt expansion of the CSC population and reduce tumor progression.	[18,102,103]

## References

[1] Singh SK, Clarke ID, Terasaki M, Bonn VE, Hawkins C (2003). Identification of a cancer stem cell in human brain tumors. Cancer Res.

[2] Singh SK, Hawkins C, Clarke ID, Squire JA, Bayani J (2004). Identification of human brain tumour initiating cells. Nature.

[3] Chen J, Li Y, Yu TS, McKay RM, Burns DK (2012). A restricted cell population propagates glioblastoma growth after chemotherapy. Nature.

[4] Ben-Porath I, Thomson MW, Carey VJ, Ge R, Bell GW (2008). An embryonic stem cell-like gene expression signature in poorly differentiated aggressive human tumors. Nat Genet.

[5] Dahan P, Martinez Gala J, Delmas C, Monferran S, Malric L (2014). Ionizing radiations sustain glioblastoma cell dedifferentiation to a stem-like phenotype through survivin: possible involvement in radioresistance. Cell Death Dis.

[6] Auffinger B, Tobias AL, Han Y, Lee G, Guo D (2014). Conversion of differentiated cancer cells into cancer stem-like cells in a glioblastoma model after primary chemotherapy. Cell Death Differ.

[7] Pistollato F, Abbadi S, Rampazzo E, Persano L, Puppa AD (2010). Intratumoral hypoxic gradient drives stem cells distribution and MGMT expression in glioblastoma. Stem Cells.

[8] Heddleston JM, Li Z, McLendon RE, Hjelmeland AB, Rich JN (2009). The hypoxic microenvironment maintains glioblastoma stem cells and promotes reprogramming towards a cancer stem cell phenotype. Cell Cycle.

[9] Iliopoulos D, Hirsch HA, Wang G, Struhl K (2011). Inducible formation of breast Cancer Stem Cells and their dynamic equilibrium with non-stem cancer cells via IL6 secretion. Proc Natl Acad Sci U S A.

[10] Chaffer CL, Brueckmann I, Scheel C, Kaestli AJ, Wiggins PA (2011). Normal and neoplastic nonstem cells can spontaneously convert to a stem-like state. Proc Natl Acad Sci U S A.

[11] Easwaran H, Tsai HC, Baylin SB (2014). Cancer epigenetics: tumor heterogeneity, plasticity of stem-like states, and drug resistance. Mol Cell.

[12] Nowell PC (1976). The clonal evolution of tumor cell populations. Science.

[13] Greaves M, Maley CC (2012). Clonal evolution in cancer. Nature.

[14] Shlush LI, Hershkovitz D (2015). Clonal evolution models of tumor heterogeneity. Am Soc Clin Oncol Educ Book.

[15] Bailar JC, Gornik HL (1997). Cancer undefeated. N Engl J Med.

[16] Gotoh N (2009). Control of stemness by fibroblast growth factor signaling in stem cells and Cancer Stem Cells. Curr Stem Cell Res Ther.

[17] Kreso A, Dick JE (2014). Evolution of the cancer stem cell model. Cell Stem Cell.

[18] Plaks V, Kong N, Werb Z (2015). The cancer stem cell niche: how essential is the niche in regulating stemness of tumor cells?. Cell Stem Cell.

[19] Vlashi E, Pajonk F (2015). Cancer Stem Cells, cancer cell plasticity and radiation therapy. Semin Cancer Biol.

[20] Anton K, Baehring JM, Mayer T (2012). Glioblastoma multiforme: overview of current treatment and future perspectives. Hematol Oncol Clin North Am.

[21] Stupp R, Hegi ME, Mason WP, van den Bent MJ, Taphoorn MJ (2009). Effects of radiotherapy with concomitant and adjuvant temozolomide versus radiotherapy alone on survival in glioblastoma in a randomised phase III study: 5-year analysis of the EORTC-NCIC trial. Lancet Oncol.

[22] Meacham CE, Morrison SJ (2013). Tumour heterogeneity and cancer cell plasticity. Nature.

[23] Islam F, Gopalan V, Smith RA, Lam AK (2015). Translational potential of Cancer Stem Cells : A review of the detection of Cancer Stem Cells and their roles in cancer recurrence and cancer treatment. Exp Cell Res.

[24] Murphy GF, Wilson BJ, Girouard SD, Frank NY, Frank MH (2014). Stem cells and targeted approaches to melanoma cure. Mol Aspects Med.

[25] Laga AC, Murphy GF (2010). Cellular heterogeneity in vertical growth phase melanoma. Arch Pathol Lab Med.

[26] Hendrix MJ, Seftor EA, Hess AR, Seftor RE (2003). Vasculogenic mimicry and tumour-cell plasticity: lessons from melanoma. Nat Rev Cancer.

[27] Monzani E, Facchetti F, Galmozzi E, Corsini E, Benetti A (2007). Melanoma contains CD133 and ABCG2 positive cells with enhanced tumourigenic potential. Eur J Cancer.

[28] Maniotis AJ, Folberg R, Hess A, Seftor EA, Gardner LM (1999). Vascular channel formation by human melanoma cells in vivo and in vitro: vasculogenic mimicry. Am J Pathol.

[29] Frank NY, Margaryan A, Huang Y, Schatton T, Waaga-Gasser AM (2005). ABCB5-mediated doxorubicin transport and chemoresistance in human malignant melanoma. Cancer Res.

[30] Frank NY, Schatton T, Frank MH (2010). The therapeutic promise of the cancer stem cell concept. J Clin Invest.

[31] Knudson AG (1971). Mutation and cancer: statistical study of retinoblastoma. Proc Natl Acad Sci U S A.

[32] Cavenee WK, Dryja TP, Phillips RA, Benedict WF, Godbout R (1983). Expression of recessive alleles by chromosomal mechanisms in retinoblastoma. Nature.

[33] Shackleton M, Quintana E, Fearon ER, Morrison SJ (2009). Heterogeneity in cancer: Cancer Stem Cells versus clonal evolution. Cell.

[34] Marjanovic ND, Weinberg RA, Chaffer CL (2013). Cell plasticity and heterogeneity in cancer. Clin Chem.

[35] Chen R, Nishimura MC, Bumbaca SM, Kharbanda S, Forrest WF (2010). A hierarchy of self-renewing tumor-initiating cell types in glioblastoma. Cancer Cell.

[36] Ferguson LR, Chen H, Collins AR, Connell M, Damia G (2015). Genomic instability in human cancer: Molecular insights and opportunities for therapeutic attack and prevention through diet and nutrition. Semin Cancer Biol.

[37] Ohanna M, Cheli Y, Bonet C, Bonazzi VF, Allegra M (2013). Secretome from senescent melanoma engages the STAT3 pathway to favor reprogramming of naive melanoma towards a tumor-initiating cell phenotype. Oncotarget.

[38] Giannoni E, Bianchini F, Masieri L, Serni S, Torre E (2010). Reciprocal activation of prostate cancer cells and cancer-associated fibroblasts stimulates epithelial-mesenchymal transition and cancer stemness. Cancer Res.

[39] Yang G, Quan Y, Wang W, Fu Q, Wu J (2012). Dynamic equilibrium between Cancer Stem Cells and non-stem cancer cells in human SW620 and MCF-7 cancer cell populations. Br J Cancer.

[40] Mukherjee S, Kong J, Brat DJ (2015). Cancer stem cell division: when the rules of asymmetry are broken. Stem Cells Dev.

[41] Cherciu I, Barbalan A, Pirici D, Margaritescu C, Saftoiu A (2014). Stem cells, colorectal cancer and cancer stem cell markers correlations. Curr Health Sci J.

[42] Filip S, Mokry J, English D, Vojacek J (2005). Stem cell plasticity and issues of stem cell therapy. Folia Biol (Praha).

[43] Campos-Sanchez E, Cobaleda C (2015). Tumoral reprogramming: Plasticity takes a walk on the wild side. Biochim Biophys Acta.

[44] Hjelmeland AB, Wu Q, Heddleston JM, Choudhary GS, MacSwords J (2011). Acidic stress promotes a glioma stem cell phenotype. Cell Death Differ.

[45] Balkwill FR, Capasso M, Hagemann T (2012). The tumor microenvironment at a glance. J Cell Sci.

[46] Ye J, Wu D, Wu P, Chen Z, Huang J (2014). The cancer stem cell niche: cross talk between Cancer Stem Cells and their microenvironment. Tumour Biol.

[47] Oskarsson T, Batlle E, Massague J (2014). Metastatic stem cells: sources, niches, and vital pathways. Cell Stem Cell.

[48] Sheng XR, Brawley CM, Matunis EL (2009). Dedifferentiating spermatogonia outcompete somatic stem cells for niche occupancy in the Drosophila testis. Cell Stem Cell.

[49] Barroca V, Lassalle B, Coureuil M, Louis JP, Le Page F (2009). Mouse differentiating spermatogonia can generate germinal stem cells in vivo. Nat Cell Biol.

[50] Cobaleda C, Schebesta A, Delogu A, Busslinger M (2007). Pax5: the guardian of B cell identity and function. Nat Immunol.

[51] Cobaleda C, Jochum W, Busslinger M (2007). Conversion of mature B cells into T cells by dedifferentiation to uncommitted progenitors. Nature.

[52] Greenow K, Clarke AR (2012). Controlling the stem cell compartment and regeneration in vivo: the role of pluripotency pathways. Physiol Rev.

[53] Patrawala L, Calhoun T, Schneider-Broussard R, Li H, Bhatia B (2006). Highly purified CD44+ prostate cancer cells from xenograft human tumors are enriched in tumorigenic and metastatic progenitor cells. Oncogene.

[54] Mani SA, Guo W, Liao MJ, Eaton EN, Ayyanan A (2008). The epithelialmesenchymal transition generates cells with properties of stem cells. Cell.

[55] Briegel KJ (2006). Embryonic transcription factors in human breast cancer. IUBMB Life.

[56] Yang J, Mani SA, Donaher JL, Ramaswamy S, Itzykson RA (2004). Twist, a master regulator of morphogenesis, plays an essential role in tumor metastasis. Cell.

[57] Oft M, Akhurst RJ, Balmain A (2002). Metastasis is driven by sequential elevation of H-ras and Smad2 levels. Nat Cell Biol.

[58] Hartwell KA, Muir B, Reinhardt F, Carpenter AE, Sgroi DC (2006). The Spemann organizer gene, Goosecoid, promotes tumor metastasis. Proc Natl Acad Sci U S A.

[59] Rhim AD, Mirek ET, Aiello NM, Maitra A, Bailey JM (2012). EMT and dissemination precede pancreatic tumor formation. Cell.

[60] Visvader JE (2011). Cells of origin in cancer. Nature.

[61] Mikheeva SA, Mikheev AM, Petit A, Beyer R, Oxford RG (2010). TWIST1 promotes invasion through mesenchymal change in human glioblastoma. Mol Cancer.

[62] Bennewith KL, Durand RE (2004). Quantifying transient hypoxia in human tumor xenografts by flow cytometry. Cancer Res.

[63] Brurberg KG, Thuen M, Ruud EB, Rofstad EK (2006). Fluctuations in pO2 in irradiated human melanoma xenografts. Radiat Res.

[64] Ghattass K, Assah R, El-Sabban M, Gali-Muhtasib H (2013). Targeting hypoxia for sensitization of tumors to radio- and chemotherapy. Curr Cancer Drug Targets.

[65] Kitamura T, Qian BZ, Pollard JW (2015). Immune cell promotion of metastasis. Nat Rev Immunol.

[66] Dineen SP, Lynn KD, Holloway SE, Miller AF, Sullivan JP (2008). Vascular endothelial growth factor receptor 2 mediates macrophage infiltration into orthotopic pancreatic tumors in mice. Cancer Res.

[67] Stockmann C, Doedens A, Weidemann A, Zhang N, Takeda N (2008). Deletion of vascular endothelial growth factor in myeloid cells accelerates tumorigenesis. Nature.

[68] Charles N, Ozawa T, Squatrito M, Bleau AM, Brennan CW (2010). Perivascular nitric oxide activates notch signaling and promotes stem-like character in PDGF-induced glioma cells. Cell Stem Cell.

[69] Hamerlik P, Lathia JD, Rasmussen R, Wu Q, Bartkova J (2012). Autocrine VEGF-VEGFR2-Neuropilin-1 signaling promotes glioma stem-like cell viability and tumor growth. J Exp Med.

[70] Beck B, Driessens G, Goossens S, Youssef KK, Kuchnio A (2011). A vascular niche and a VEGF-Nrp1 loop regulate the initiation and stemness of skin tumours. Nature.

[71] Friedman HS, Prados MD, Wen PY, Mikkelsen T, Schiff D (2009). Bevacizumab alone and in combination with irinotecan in recurrent glioblastoma. J Clin Oncol.

[72] de Groot JF, Fuller G, Kumar AJ, Piao Y, Eterovic K (2010). Tumor invasion after treatment of glioblastoma with bevacizumab: radiographic and pathologic correlation in humans and mice. Neuro Oncol.

[73] Schnegg CI, Yang MH, Ghosh SK, Hsu MY (2015). Induction of Vasculogenic Mimicry Overrides VEGF-A Silencing and Enriches Stem-like Cancer Cells in Melanoma. Cancer Res.

[74] Gerweck LE, Seetharaman K (1996). Cellular pH gradient in tumor versus normal tissue: potential exploitation for the treatment of cancer. Cancer Res.

[75] Kallinowski F, Vaupel P (1988). pH distributions in spontaneous and isotransplanted rat tumours. Br J Cancer.

[76] Gatenby RA, Gillies RJ (2004). Why do cancers have high aerobic glycolysis?. Nat Rev Cancer.

[77] Chiche J, Brahimi-Horn MC, Pouyssegur J (2010). Tumour hypoxia induces a metabolic shift causing acidosis: a common feature in cancer. J Cell Mol Med.

[78] Bao S, Wu Q, McLendon RE, Hao Y, Shi Q (2006). Glioma stem cells promote radioresistance by preferential activation of the DNA damage response. Nature.

[79] Li X, Lewis MT, Huang J, Gutierrez C, Osborne CK (2008). Intrinsic resistance of tumorigenic breast cancer cells to chemotherapy. J Natl Cancer Inst.

[80] Mitra A, Mishra L, Li S (2015). EMT, CTCs and CSCs in tumor relapse and drug-resistance. Oncotarget.

[81] Liang H, Deng L, Chmura S, Burnette B, Liadis N (2013). Radiation-induced equilibrium is a balance between tumor cell proliferation and T cell-mediated killing. J Immunol.

[82] Huang R, Wang G, Song Y, Tang Q, You Q (2015). Colorectal cancer stem cell and chemoresistant colorectal cancer cell phenotypes and increased sensitivity to Notch pathway inhibitor. Mol Med Rep.

[83] Thakur R, Trivedi R, Rastogi N, Singh M, Mishra DP (2015). Inhibition of STAT3, FAK and Src mediated signaling reduces cancer stem cell load, tumorigenic potential and metastasis in breast cancer. Sci Rep.

[84] Liu H, Lv L, Yang K (2015). Chemotherapy targeting Cancer Stem Cells. Am J Cancer Res.

[85] Cojoc M, Mabert K, Muders MH, Dubrovska A (2015). A role for Cancer Stem Cells in therapy resistance: cellular and molecular mechanisms. Semin Cancer Biol.

[86] Paez D, Labonte MJ, Bohanes P, Zhang W, Benhanim L (2012). Cancer dormancy: a model of early dissemination and late cancer recurrence. Clin Cancer Res.

[87] Szakacs G, Paterson JK, Ludwig JA, Booth-Genthe C, Gottesman MM (2006). Targeting multidrug resistance in cancer. Nat Rev Drug Discov.

[88] Jackson SP (2009). The DNA-damage response: new molecular insights and new approaches to cancer therapy. Biochem Soc Trans.

[89] Cheung-Ong K, Giaever G, Nislow C (2013). DNA-damaging agents in cancer chemotherapy: serendipity and chemical biology. Chem Biol.

[90] Brandsma I, Gent DC (2012). Pathway choice in DNA double strand break repair: observations of a balancing act. Genome Integr.

[91] Ochi T, Blackford AN, Coates J, Jhujh S, Mehmood S (2015). DNA repair. PAXX, a paralog of XRCC4 and XLF, interacts with Ku to promote DNA double-strand break repair. Science.

[92] Rivera M, Wu Q, Hamerlik P, Hjelmeland AB, Bao S (2015). Acquisition of meiotic DNA repair regulators maintain genome stability in glioblastoma. Cell Death Dis.

[93] Ezashi T, Das P, Roberts RM (2005). Low O2 tensions and the prevention of differentiation of hES cells. Proc Natl Acad Sci U S A.

[94] Jasin M, Rothstein R (2013). Repair of strand breaks by homologous recombination. Cold Spring Harb Perspect Biol.

[95] Al-Kaabi MM, Alshareeda AT, Jerjees DA, Muftah AA, Green AR (2015). Checkpoint kinase1 (CHK1) is an important biomarker in breast cancer having a role in chemotherapy response. Br J Cancer.

[96] Al-Assar O, Mantoni T, Lunardi S, Kingham G, Helleday T (2011). Breast cancer stem-like cells show dominant homologous recombination due to a larger S-G2 fraction. Cancer Biol Ther.

[97] Woodward WA, Chen MS, Behbod F, Alfaro MP, Buchholz TA (2007). WNT/beta-catenin mediates radiation resistance of mouse mammary progenitor cells. Proc Natl Acad Sci U S A.

[98] Diehn M, Cho RW, Lobo NA, Kalisky T, Dorie MJ (2009). Association of reactive oxygen species levels and radioresistance in Cancer Stem Cells. Nature.

[99] Alagpulinsa DA, Ayyadevara S, Reis RJS (2014). A Small-Molecule Inhibitor of RAD51 Reduces Homologous Recombination and Sensitizes Multiple Myeloma Cells to Doxorubicin. Front Oncol.

[100] Ward A, Khanna KK, Wiegmans AP (2015). Targeting homologous recombination, new pre-clinical and clinical therapeutic combinations inhibiting RAD51. Cancer Treat Rev.

[101] Normand A, Riviere E, Renodon-Corniere A (2014). Identification and characterization of human Rad51 inhibitors by screening of an existing drug library. Biochem Pharmacol.

[102] Li Z, Bao S, Wu Q, Wang H, Eyler C (2009). Hypoxia-inducible factors regulate tumorigenic capacity of glioma stem cells. Cancer Cell.

[103] Gordan JD, Bertout JA, Hu CJ, Diehl JA, Simon MC (2007). HIF-2alpha promotes hypoxic cell proliferation by enhancing c-myc transcriptional activity. Cancer Cell.

[104] Gu JW, Rizzo P, Pannuti A, Golde T, Osborne B (2012). Notch signals in the endothelium and cancer "stem-like" cells: opportunities for cancer therapy. Vasc Cell.

[105] Evans JM, Donnelly LA, Emslie-Smith AM, Alessi DR, Morris AD (2005). Metformin and reduced risk of cancer in diabetic patients. BMJ.

[106] Mezencev R, Wang L, McDonald JF (2012). Identification of inhibitors of ovarian cancer stem-like cells by high-throughput screening. J Ovarian Res.

[107] Germain AR, Carmody LC, Nag PP, Morgan B, Verplank L (2013). Cinnamides as selective small-molecule inhibitors of a cellular model of breast Cancer Stem Cells. Bioorg Med Chem Lett.

[108] Liu P, Kumar IS, Brown S, Kannappan V, Tawari PE (2013). Disulfiram targets cancer stem-like cells and reverses resistance and cross-resistance in acquired paclitaxel-resistant triple-negative breast cancer cells. Br J Cancer.

[109] Sun Y, Hu J, Zhou L, Pollard SM, Smith A (2011). Interplay between FGF2 and BMP controls the self-renewal, dormancy and differentiation of rat neural stem cells. J Cell Sci.

[110] Zhou Z, Sun L, Wang Y, Wu Z, Geng J (2011). Bone morphogenetic protein 4 inhibits cell proliferation and induces apoptosis in glioma stem cells. Cancer Biother Radiopharm.

[111] Liu B, Chen Q, Tian D, Wu L, Dong H (2013). BMP4 reverses multidrug resistance through modulation of BCL-2 and GDNF in glioblastoma. Brain Res.

[112] Chirasani SR, Sternjak A, Wend P, Momma S, Campos B (2010). Bone morphogenetic protein-7 release from endogenous neural precursor cells suppresses the tumourigenicity of stem-like glioblastoma cells. Brain.

[113] Lombardo Y, Scopelliti A, Cammareri P, Todaro M, Iovino F (2011). Bone morphogenetic protein 4 induces differentiation of colorectal Cancer Stem Cells and increases their response to chemotherapy in mice. Gastroenterology.

[114] Piccirillo SG, Vescovi AL (2006). Bone morphogenetic proteins regulate tumorigenicity in human glioblastoma stem cells. Ernst Schering Found Symp Proc.

[115] Li B (2008). Bone morphogenetic protein-Smad pathway as drug targets for osteoporosis and cancer therapy. Endocr Metab Immune Disord Drug Targets.

[116] Persano L, Pistollato F, Rampazzo E, Puppa AD, Abbadi S (2012). BMP2 sensitizes glioblastoma stem-like cells to Temozolomide by affecting HIF-1alpha stability and MGMT expression. Cell Death Dis.

[117] Rahman M, Azari H, Deleyrolle L, Millette S, Zeng H (2013). Controlling tumor invasion: bevacizumab and BMP4 for glioblastoma. Future Oncol.

[118] Buijs JT, van der Horst G, van den Hoogen C, Cheung H, de Rooij B (2012). The BMP2/7 heterodimer inhibits the human breast cancer stem cell subpopulation and bone metastases formation. Oncogene.

[119] Ye L, Bokobza SM, Jiang WG (2009). Bone morphogenetic proteins in development and progression of breast cancer and therapeutic potential (review). Int J Mol Med.

[120] Czerwinska P, Kaminska B (2015). Regulation of breast cancer stem cell features. Contemp Oncol (Pozn).

[121] Gonzalez-Gomez P, Anselmo NP, Mira H (2014). BMPs as therapeutic targets and biomarkers in astrocytic glioma. Biomed Res Int.

